# Allelopathy and Allelochemicals of *Imperata cylindrica* as an Invasive Plant Species

**DOI:** 10.3390/plants11192551

**Published:** 2022-09-28

**Authors:** Hisashi Kato-Noguchi

**Affiliations:** Department of Applied Biological Science, Faculty of Agriculture, Kagawa University, Miki 761-0795, Kagawa, Japan; kato.hisashi@kagawa-u.ac.jp

**Keywords:** allelochemical, decomposition, invasive species, monospecific stand, mycorrhizal colonization, phytotoxicity, rhizobium nodulation

## Abstract

*Imperata cylindrica* is native to Southeast Asia and East Africa and has become naturalized in humid tropics, subtropics and warmer temperate zones of the world. The species is one of the top ten worst weeds in the worlds and is listed among the world’s top 100 worst invasive alien species. It is an aggressive colonizer and forms large monospecific stands in several countries. Possible evidence of the allelopathy of *I. cylindrica* has been accumulated in the literature over three decades. The extracts, leachates, root exudates, decomposing residues and rhizosphere soil of *I. cylindrica* were found to suppress the germination and growth of several plant species, including woody plant species, and to reduce their rhizobium nodulation and mycorrhizal colonization. Several allelochemicals, such as fatty acids, terpenoids, simple phenolics, benzoic acids, phenolic acids, phenolic aldehydes, phenylpropanoids, flavonoids, quinones and alkaloids, were also found in the extracts, leachates, root exudates and/or growth medium of *I. cylindrica*. These observations suggest that allelochemicals may be synthesized in *I. cylindrica* and released into the rhizosphere soil and surrounding environments either by the leachates, root exudation or decomposition process of plant parts, and certain allelochemicals may contribute to the alteration of the microbial community, including rhizobia and mycorrhizal fungi, suppressing the regeneration process of native plant species through the inhibition of their germination and growth. Therefore, the allelopathy of *I. cylindrica* may support its invasiveness, naturalization and formation of large monospecific stands. This is the first review article focusing on the allelopathy of *I. cylindrica*.

## 1. Introduction

**Figure 1:**
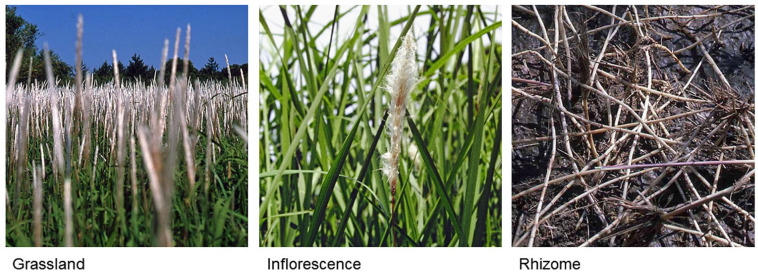
*Imperata cylindrica*. Photos were kindly provided by Dr. T. Tominaga.

*Imperata cylindrica* (L.) Beauv., belonging to Poaceae, is a C_4_ perennial rhizomatous grass species (Figure 1). This species has an extensive rhizome network. The rhizome development of *I. cylindrica* occurs 3–12 weeks after germination, beginning with downward growth. When the rhizome develops cataphylls, it grows horizontally, and the tip of the rhizome begins to turn upward between the fifth and sixth leaf stages. Then, a secondary shoot and rhizome arise from the apical bud and subapical bud of the first rhizome, respectively. The secondary rhizome produces the third shoot and rhizome, continuing the following shoot and rhizome formations [1,2,3,4,5]. It was reported that one plant produced 31 rhizomes in 10 months, with a total rhizome length of 12 m [6]. The shoots form loose to compact tufts. Several leaf sheaths are often tightly rolled together, forming a cylindrical culm. *I. cylindrica* is virtually stemless, except for the flowering stalks. The leaves are flat, slender (4–10 mm in wide) and linear–lanceolate (15–150 cm in length, depending on the growth condition), possessing serrated margins embedded with sharp silica crystals. Stomata are present on both surfaces of the leaves [7,8,9,10].

*I. cylindrica* is native to Southeast Asia and East Africa and has become naturalized in humid tropics, subtropics and warmer temperate zones of the world [1,11,12,13,14]. The species is considered one of the top ten worst weeds in the world and is listed among the world’s top 100 worst invasive alien species [1,12,13]. *I. cylindrica* thrives in the 10 southeastern states of the USA, the Mediterranean region, northern Africa to the Middle East, tropical and subtropical Asia, Australia and the Pacific Islands, covering more than 500 million hectares worldwide [3,12,13,15,16,17]. Very large monospecific stands of *I. cylindrica* of more than 10,000 hectares in size, known as mega grasslands, are often observed in Indonesia as a climax species [18,19]. Large monospecific stands are also observed in Africa from Egypt to Ethiopia [1]. The species grows between latitudes 45° N (e.g., Japan) and 45° S (New Zealand) and from sea level to more than 2000 m in elevation [20,21].

The characteristics of life history, such as high reproduction and growth rates, competitive ability and phenotypic plasticity, of the plants are important for the naturalization of invasive plants into non-native ranges [22,23,24,25]. *I. cylindrica* is also a prolific seed producer, although its flowering is variable, depending on the environmental conditions. The inflorescence of *I. cylindrica* is 10–20 cm in length, with approximately 400 spikelets, which are 3–6 cm in length and covered with short, white hairs [5,7,8]. (Figure 1). A single plant produces 500–3000 seeds, which are contained in the spikelets. The movement of the seeds was reported to generally span 15 m, traveled distances of as much as 24 km with the spikelets through wind dispersal [1,26,27]. The germination rate is 30–98%, depending on the conditions [8,28,29].

Vegetative reproduction maintains local stands of *I. cylindrica* and contributes to spread the plant in its vicinity. When rhizome pieces are dispersed by human activities and other disturbing factors, such as a hurricane, the establishment of new populations arises from rhizome pieces. The regeneration of *I. cylindrica* can occur within a small amount of rhizomes—as much as 0.1 g [30]. *I. cylindrica* rhizomes are also very fertile [31]. It was estimated that *I. cylindrica* produces 6 tons of rhizome biomass per hectare, with more than 4.5 million shoots and 10 metric tons of leaf biomass [32]. Terry et al. [33] also estimated a rhizome biomass of 40 tons per hectare. Over the course of 6 weeks, rhizomes were reported to develop 350 shoots, covering an area of 4 m^2^ [34]. The extensive growth of *I. cylindrica* results dense mats of rhizomes in the soil, forming thick monospecific patches [32,33] (Figure 1).

*I. cylindrica* is an excellent competitor for nutrients. Its ability to absorb phosphorus, nitrogen and potassium is higher than that of native pine savanna plants, including *Pinus taeda* L. [35,36,37]. The species was reported to reduce the availability of water and nitrogen in surrounding soil, suppressing the establishment of juvenile *P. taeda* by 50% [38].

*I. cylindrica* has also been reported to increase wildfire occurrence and maximum wildfire temperatures [39,40]. High-temperature fire increases the mortality of native herbaceous and juvenile woody plant spices. Because *I. cylindrica* allocates a significant amount of biomass (over 60%) in below-ground rhizomes, it is able to regenerate quickly from its rhizomes after fires [37,39,41,42]. Therefore, frequent intense fires may result in the conversion of native ecosystems of herbaceous and juvenile woody plants into *I. cylindrica*-dominated grasslands.

The phenotypic plasticity of *I. cylindrica* is high, and the species has adapted to a wide range of environmental conditions, such as various soil types, pH values (4.4 to 8.0), organic matter (0.9 to 5%) and nutrients (P; 6 to190 kg/ha, K; 46 to 734 kg/ha) [43]. It thrives in tropical, subtropical and warm temperate areas, where annual rainfall is 750–5000 mm [7]. Its characteristics in terms of floral traits (such as spikelets, anthers and glumes) and leaf traits (such as length and width) vary within the species [27,44,45]. The species can be separated into five major varieties based on growth and morphological characteristics, as well as geographic origin: *I. cylindrica* var. *europa* (2*n* = 40), *major* (2*n* = 20), *Africana* (2*n* = 60), *latifolia* (not available) and *condensate* (not available). The genetic diversity of *I. cylindrica* in 676 samples from seven southern US states was determined using amplified fragment length polymorphisms, with two genetic lineages identified [46]. Random amplified polymorphic DNA analysis conducted in Florida showed that the species was highly outcrossed [8]. Hybridization may have facilitated the expansion of the species.

The interactions of these invasive plants with natural enemies, such as herbivores and pathogens, are critical for their naturalization. [23,24,47]. Multiple insect predators and fungal pathogens have been identified in *I. cylindrica* stands. However, none of them caused the extensive damage to the population of *I. cylindrica* [21]. In addition, methanol extracts of shoots, roots and inflorescences of *I. cylindrica* showed antifungal activity against the plant pathogen *Macrophomina phaseolina* (Tassi) Goid. [48].

The chemical interactions of the invasive plants with native plants are also crucial [24,49,50]. Several observations suggest that some invasive plants are allelopathic and that their allelochemicals are toxic to native plant species in the invasive ranges [51,52]. Therefore, allelopathy may among the factors allowing the invasive plants to establish their habitats and naturalize within the invasive ranges [52]. Allelopathy is the chemical interaction between a donor plant and receiver plants through the specific secondary metabolites defined as allelochemicals [53]. Available data from a number of observations under field and laboratory conditions suggest that *I. cylindrica* is also allelopathic*,* and possible evidence for the compounds involved in this allelopathy has been accumulated. However, no review papers have been published focusing on the allelopathy of *I. cylindrica*. Therefore, in this review, we provide an overview of the allelopathy and allelochemicals of the *I. cylindrica* and discuss the possible involvement of allelopathy in the invasiveness of the species.

## 2. Effects of *I. cylindrica* on Microbial Community

Several investigations under field and greenhouse conditions have showed that *I. cylindrica* altered the microbial community in the rhizosphere soil and affected the growth of several plant species, including crop plants. The decomposition process of an *I. cylindrica* residue was reported to be significantly faster than that of a local native grass species, *Andropogon glomeratus* (Walt.) Britton, Sterns & Poggenb. *I. cylindrica* was reported to affect the microbial flora in the soil of invaded areas and enhance the decomposition process of the plant residue [54]. The enhanced decomposition process of the plant residue may enable increasing nutrient cycling and availability. Although there are many possible explanations for the alteration of the microbial community in the soil, certain compounds of *I. cylindrica* may be involved in this alteration.

The abundance of mycorrhizal colonization and biomass of fine feeder roots of *Pinus taeda* L. was significantly suppressed in *I. cylindrica*-infested plantations compared with plantations not infested by *I. cylindrica*. The diversity of understory vegetation in pine plantations was also reduced by the presence of *I. cylindrica* [55]. The invasion of *I. cylindrica* into a *Pinus palustris* Mill. community caused significant loss of native plant species and reduced the distinctiveness of the native flora [56]. Arbuscular mycorrhizal colonization of *Mesicago denticulata* Greene and *Trifolium resupinatum* L. was decreased in *I. cylindrica*-infested fields compared with non-infested fields [57]. The mycorrhizal colonization of some weed species was also suppressed by the presence of *I. cylindrica* [58]. In addition, the rhizobium nodulation, nitrogen fixation and root growth of *Melilotus parviflora* Desf. were suppressed when *M. parviflora* was grown together with *I. cylindrica*. The populations of some fungi, such as *Aspergillus spp*., also differed between *I. cylindrica*-infested soil and non-infested soil [59]. When *I. cylindrica* was grown with *Zea mays* L. or *Sorghum bicolor* (L.) Moench for 8 weeks, the biomass of *I. cylindrica* was not affected by the presence of *Z. mays* and *S. bicolor.* However, the biomass of *Z. mays* and *S. bicolor* was significantly reduced by the presence of *I. cylindrica* even under the conditions that eliminated possible nutrient competition [60]. These observations suggest that *I. cylindrica* may suppress the mycorrhizal colonization and rhizobium nodulation in native plant species, leading to the growth inhibition of these plant species. Although some authors found no direct evidence of allelopathy, they suggested the involvement of *I. cylindrica* allelopathy in the suppression of mycorrhizal colonization and rhizobium nodulation [54,58,59,60].

## 3. Allelopathy of *I. cylindrica*

Allelochemicals are synthesized in certain plant parts and released into the vicinity of the plants either by rainfall leachates and volatilization from the plants, root exudation or decomposition of plant litter and residues [61,62,63]. The allelopathic activity of leachates, residues and plant extracts of *I. cylindrica* was evaluated over three decades (Table 1). Those observations suggest that *I. cylindrica* may synthesize some allelochemicals and release them into the neighboring environments either by rainfall leachates, exudation or the decomposition process of the plants.

### 3.1. Plant Leachates and Exudates

Water was sprinkled onto the chopped leaves of *I. cylindrica* as an artificial rain, and dripped water from the leaves was pumped up and sprinkled again onto the leaves. After 11 days of water circulation, dripped water was collected as artificial rainfall leachates and evaluated in terms of its allelopathic activity. The leachates suppressed the growth of *Brachiaria mutica* (Forssk.) Stapf and *Digitaria decumbens* Stent but not *I. cylindrica* itself [64]. Crushed dry shoots and roots of *I. cylindrica* were placed onto a large funnel containing a sheet of filter paper, which was kept in the open air and exposed to natural rainfall. Collected rainfall leachates from the crushed shoots and roots inhibited the germination of *Dichanthium annulatum* (Forssk.) Stapf and the radical growth of *D. annulatum, Chrysopogon montanus* Trin., *Medicago polymorpha* L. and *Pinus roxburghii* Sarg. [65].

*I. cylindrica* was grown in pots filled with a fine sand and irrigated with water twice a week, and drain water from holes at bottom of the pots was collected as *I. cylindrica* leachates. The leachates suppressed the aboveground biomass of *Aristida stricta* Michex. *var. beyrichiana* by 35.7%, its total root length by 24.9% and its mycorrhizal colonization by 23.5%. It also suppressed mycorrhizal colonization of *Pinus elliottii* Engelm by 19.5% [66]. The collected leachates may also contain its root exudates. *I. cylindrica* was grown in an agar medium for 7 day, and the medium was centrifuged after removing the plants. The resulting supernatant, which may contain root exudates of *I. cylindrica*, inhibited the roots and shoots growth of *Echinochloa crus-galli* (L.) Beauv. [67]. Soil obtained in *I. cylindrica*-dominated areas was extracted with water. The soil extracts inhibited the germination of *Dichanthium annulatum* (Forssk.) Stapf and *Chrysopogon montanus* Trin., as well as the radical growth of *Setaria italica* P. Beauv., *D. annulatum, C. montanus*, *Medicago polymorpha* L. and *Pinus roxburghii* Sarg. [65]. These observations suggest that the leachates and root exudates may contain allelochemicals, causing the growth inhibition and suppression of mycorrhizal colonization. These observations also imply that certain allelochemicals may be released from the leaves and below-ground parts of *I. cylindrica* into the rhizosphere soil or neighboring environments as plant leachates and exudates.

### 3.2. Plant Residues

The leaves of *I. cylindrica* were mixed with soil (0 g, 1.6 g and 3.2 g leaves per 100 g soil) in plastic trays (32 × 25 × 7 cm^3^), and those trays were kept in a greenhouse with occasional watering. Weeds that emerged in the trays were identified, counted and removed every week for one year. There was no significant difference in the total number of emerged weeds among treatments. However, the total biomass of the weeds was 38.8%, and that of the control was 27.1% (0 g leaf) for the soil mixed with 1.6 g and 3.2 g leaves, respectively. The predominant weed species were *Erigeron canadensis* L. and *Portulaca oleracea* L., which were also predominant weeds in the nearest experimental crop field [68].

Foliage or rhizomes and roots of *I. cylindrica* were mixed with sterilized sand medium, and the seeds of *Sida spinosa* L., *Brachiaria ramosa* (L.) Stapf., *Echinochloa crus-galli* (L.) Beauv., *Cynodon dactylon* (L.) Pers. and *Lolium multiflorum* Lam. were sown into the mixture and incubated for 10 days and 30 days to determine the rate of the germination and growth, respectively. All treatments resulted in the suppression of the germination of *S. spinosa*, *B. ramosa*, *E. crus-galli*, *C. dactylon* and *L. multiflorum* and the growth of *S. spinosa, B. ramosa* and *E. crus-galli* [69]. Whole plants of *I. cylindrica* were cut and mixed with a sand–peat medium. Rice seeds were then sown into the medium and incubated for 38 days. The treatments resulted in a reduction in the growth of *Oryza sativa* L. and the concentration of nitrogen and phosphate in rice seedlings [70].

Tillers, roots or rhizomes of *I. cylindrica* were incorporated into a soil mixture of sandy loam and sand and kept for 4 weeks. The *I. cylindrica*-incorporated soil mixtures were then extracted with water. The extracts of all soil mixtures inhibited the growth of *Trifolium subterraneum* L. radicles. However, the extract of the tiller mixture was the most effective [71]. These observations suggest that the whole plants, including leaves, roots and rhizomes, of *I. cylindrica* may contain certain allelochemicals, some of which may be released into the soil during the decomposition processes of plant residues.

### 3.3. Plant Extract

Because allelochemicals are synthesized and accumulated in some plant parts [61,62,63], many researchers have investigated the allelopathic activity of the extracts from different plant parts of *I. cylindrica*. Aqueous extracts of whole plants of *I. cylindrica* showed the inhibitory activity with respect to the germination of *Oryza sativa* L. [70]. Frequency and density of *Parthenium hysterophorus* L. was significantly lower in *I. cylindrica*-dominated areas than that in non-infested areas, and inhibition of the germination and seedling growth of *P. hysterophorus* was observed in association with treatments of the aqueous extracts of roots and shoots of *I. cylindrica* [72]. Aqueous extracts of roots and shoots of *I. cylindrica* also inhibited the germination and growth of *Cucumis sativus* L., *Lolium perenne* L. [73] and *Centrosema pubescens* Benth. [74].

Leaves, roots and rhizomes of *I. cylindrica* were soaked in water, and the obtained soaking water inhibited the germination and growth of *Raphanus sativus* L., *Brassica juncea* *var. cernua* Jorb. et Hem., *Trigonella foenum-graecum* L. and *Lycopersicon esculentum* Mill. [59]. Aqueous leaf extracts of *I. cylindrica* exhibited growth inhibitory activity on the radicals of *Solanum lycopersicum* L. (synonym; *S. lycopersicum* L.) [75]. Aqueous extracts of foliage and below-grand parts of *I. cylindrica* inhibited the germination and seedling growth of *Sida spinosa* L., *Brachiaria ramosa* (L.) Stapf., *Echinochloa crus-galli* (L.) Beauv., *Cynodon dactylon* (L.) Pers. and *Lolium multiflorum* Lam. [76]. Aqueous extracts of inflorescences, shoots and roots of *I. cylindrica* suppressed the germination and radicle growth of *Setaria italica* P. Beauv., *Dichanthium annulatum* (Forssk.) Stapf*, Chrysopogon montanus* Trin., *Medicago polymorpha* L. and *Pinus roxburghii* Sarg. [65]. It was also reported that aqueous extracts of *I. cylindrica* shoots suppressed the rhizobium nodulation and arbuscular mycorrhizal colonization of *Vigna radiata* (L.) Wilczek and *Phaseolus vulgaris* L. [77].

Aqueous methanol extracts of *I. cylindrica* rhizomes inhibited the root and shoot growth of three monocotyledonous plants, *Echinochloa crus-galli* (L.) Beauv., *Lolium multiflorum* Lam. and *Phleum pratense* L., as well as that of three dicotyledonous plants, *Lepidium sativum* L., *Lactuca sativa* L. and *Medicago sativa* L. [78]. Leaf and rhizome powder of *I. cylindrica* was extracted with methanol, and the extract was concentrated. The concentrated extract was dissolved in water and sprayed onto the plant surface of *Amaranthus spinosus* L. The treatments resulted in a significant reduction in the growth of *A. spinosus* [79]. In addition, essential oil of roots and aerial parts of *I. cylindrica* suppressed the germination and seedlings of *Lactuca sativa* L. and *Agrostis stolonifera* L. [80].

Observations reported in this section suggest that the aqueous and methanol extracts of every part of *I. cylindrica* exhibit allelopathic activity with respect to the germination, growth, rhizobium nodulation and/or mycorrhizal colonization of several plant species and probably contain water- and methanol-extractable allelochemicals, which may cause the suppression of germination, growth, mycorrhizal colonization and/or rhizobium nodulation.

## 4. Allelochemicals of *I. cylindrica*

**Figure 2:**
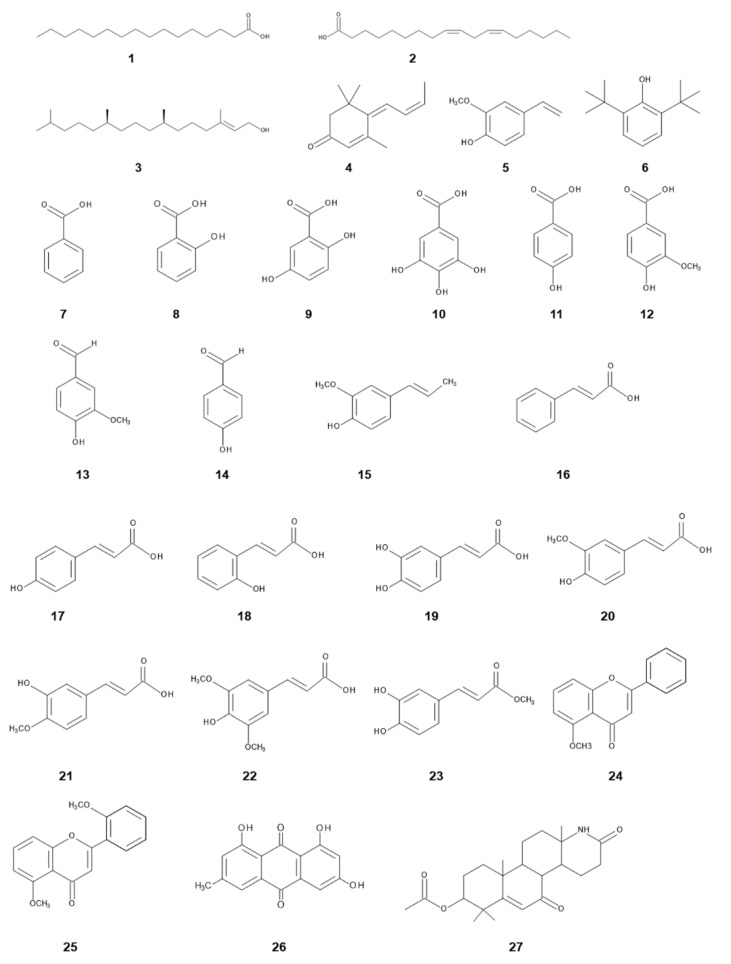
Allelochemicals identified in *Imperata cylindrica.* Compound numbers and names are listed in Table 2.

Many secondary metabolites were isolated and identified in the leachates, exudates and extracts of *I. cylindrica*, as well as its allelopathic agents (Figure 2, Table 2). *p*-Hydroxybenzoic acid and *p*-coumaric acid were identified in artificial rainfall leachates from leaves of *I. cylindrica* [64]. Benzoic acid, salicylic acid, gallic acid, cinnamic acid, caffeic acid, sinapinic acid (sinapic acid), emodin and hexadecahydro-1-azachrysen-8-yl ester were identified in the drain water from the *I. cylindrica* growth medium. However, their concentrations were 0.6–18 μM [66], which may be lower than their biologically active concentrations.

Four compounds, palmitic acid, phytol, tabanone (4,6,8-megastigmatrien-3-one) and *p*-vinylguaiacol, were identified in the essential oil obtained from roots and aerial parts of *I. cylindrica*. Phytol, palmitic acid and *p*-vinylguaiacol did not show significant growth inhibitory activity against *Lactuca sativa* L. and *Agrostis stolonifera* L. Tabanone inhibited the growth of *Lemna aequinoctialis* Welw., *L. sativa* and *Allium cepa* L. The concentrations required for 50% growth inhibition of tabanone on *L. aequinoctialis*, *L. sativa* and *A. cepa* were 0.094, 6.5 and 3.6 mM, respectively. Tabanone also caused the electrolyte leakage of the cotyledon discs of *Cucumis sativus* L. and reduced photosynthetic electron transport of the discs. However, tabanone was not found in the soil under *I. cylindrica* [80].

Three allelochemicals, methyl caffeate, 5-methoxyflavone and 5,2′-dimethoxyflavone, were isolated from the aqueous methanol extracts of *I. cylindrica* rhizomes, and these compounds significantly inhibited the root and shoot growth of *Lepidium sativum* L. The concentrations required for 50% growth inhibition of 5-methoxyflavone, 5,2′-dimethoxyflavone and methyl caffeate were 0.24, 0.079 and 1.1 mM for *L. sativum* roots, and 0.23, 0.88 and 0.59 mM for *L. sativum* shoots, respectively [78]. Benzoic acid, gentisic acid, *p*-hydroxybenzoic acid, vanillic acid, vanillin, *p*-hydroxybenzaldehyde, *p*-coumaric acid and *o*-coumaric acid were also identified in the aqueous leaf extracts [75].

A total of 36 compounds were identified: 27 compounds in the aqueous rhizome extracts of *I. cylindrica* and 24 compounds in its root exudates. Fifteen of these compounds were identified in both the extracts and exudates. Major compounds in the rhizome extracts were isoeugenol (392 μg/rhizome), isoferulic acid (289 μg/rhizome), linoleic acid (253 μg/rhizome), ferulic acid (217 μg/rhizome) and vanillin (217 μg/rhizome), and those in the root exudates were 4-acetyl-2-methoxyphenol (acetoguaiacone; 872 μg/plant) and palmitic acid (221 μg/plant). Isoeugenol (100 ppm) inhibited the growth of *Bidens pilosa* L., *Leucaena leucocephala* (Lam.) de Wit and *Echinochloa crus-galli* (L.) Beauv. Isoferulic acid inhibited the growth of *B. pilosa*, *L. leucocephala* and *E. crus-galli*. Ferulic acid, vanillin, 4-acetyl-2-methoxyphenol and palmitic acid inhibited the growth of *B. pilosa* and *L. leucocephala*. 2,4-Di-*tert*-butylphenol was also found in the rhizome extract at a concentration of 19.7 μg/rhizome. Its inhibitory activity was the most active of all compounds, followed by isoeugenol and 4-acetyl-2-methoxyphenol [67]. However, 2,4-di-*tert*-butylphenol is a synthetic phenolic antioxidant and the most widely used additive globally [81,82]. The contamination of 2,4-di-*tert*-butylphenol into the natural environment was also reported through the degradation of multiphenolic rings of synthetic phenolic antioxidants [83].

## 5. Contribution of Allelopathy of *I. cylindrica* to Its Invasiveness

*I. cylindrica* shows high reproductive ability through seeds and rhizomes; its high growth rate and highly competitive ability with native plant species is described in the “Introduction” section [1,27,31,33,36,37]. Its phenotypic plasticity is also high, and it has adapted to a wide range of soil and climate conditions [7,43]. The species alters wildfire regimes and increases mortality of native plant species. None insects, fungi or other predators cause extensive damage to *I. cylindrica* populations [21]. These characteristics may contribute to the invasiveness of *I. cylindrica* and its naturalization. Many studies also suggest that its allelopathy may contribute to its invasiveness and naturalization [5,15,54,84].

The leachates, root exudates, residues and extracts of the plant parts of *I. cylindrica* exhibit allelopathic activity against the seed germination and growth of several plant species, including native plant species (Table 1). Several allelochemicals, such as fatty acids, terpenoids, simple phenolics, benzoic acid, phenolic acids, phenolic aldehydes, phenylpropanoids, flavonoids, quinone and alkaloids, were also isolated and identified in the leachates, root exudates, essential oil and extracts of *I. cylindrica* (Table 2; Figure 2). The derivatives of cinnamic acid and benzoic acid have been found in a wide range of plants and soils. Their inhibitory activity with respect to plant growth and germination is concentration-dependent. These compounds are often mentioned as putative allelochemicals, although their levels in soil and plants are differ significantly between publications. The derivatives of cinnamic acid and benzoic acid are synthesized by the shikimic acid pathway and serve as precursors for a wide variety of important compounds, playing crucial roles in plant fitness, including plant hormones and defense compounds [85]. Cinnamic acid and benzoic acid derivatives affect cell membrane permeability and reduce the potential of ion and nutrient uptake. They also affect stomatal functions and water balance and interfere with several enzyme activities involved in major physiological processes, such as respiration, protein synthesis, phytohormone synthesis and metabolism of some other secondary metabolites [86,87,88,89]. Therefore, cinnamic acid and benzoic acid derivatives found in *I. cylindrica,* such as benzoic acid, gentisic acid, gallic acid, *p*-hydroxybenzoic acid, vanillic acid, isoeugenol, cinnamic acid, *p*-coumaric acid, caffeic acid, ferulic acid and sinapinic acid, may affect some of those physiological processes of native plant species and decrease their germination, growth and fitness. Salicylic acid is a signaling molecule involved in plant defense systems against insect and fungal attacks, as well as other stress conditions [90,91]. Tabanone was reported to reduce photosynthetic electron transport [80]. Information on the mode of action of other compounds found in *I. cylindrica* is limited.

**Figure 3:**
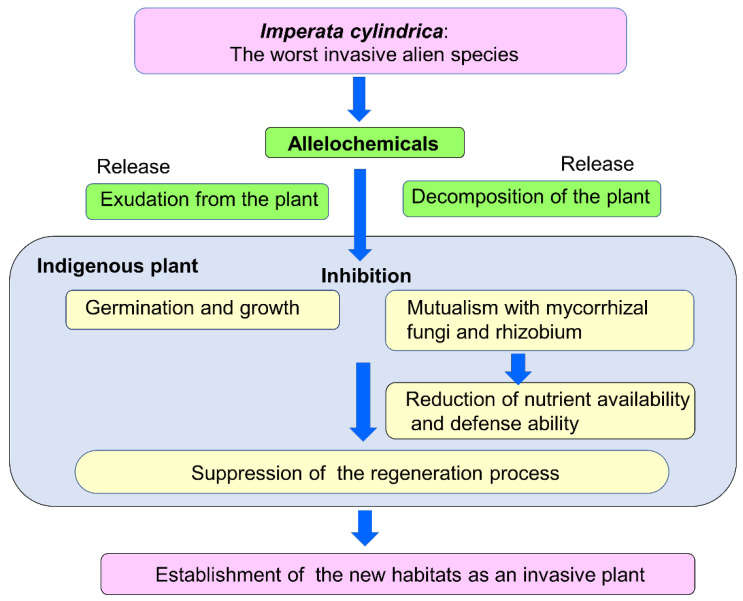
A possible scheme of *I. cylindrica* to establish the new habitats.

Recent pharmacological investigations showed that *I. cylindrica* contains secondary metabolites in many chemical classes, such as saponins, flavonoids, phenols and glycosides. in its rhizomes and other parts of the plants. Some of those compounds exhibited anti-inflammatory, antitumor, antibacterial, hematuria and diuretic activity in previous studies [92,93,94,95,96,97,98]. Although the allelopathic activity of most identified compounds has not yet been reported to date, a number of secondary metabolites in the invasive plants showed multiple effects, such as antiherbivore, antifungal and allelopathic activity [24,51,99]. Therefore, some of those compounds may exhibit growth inhibitory activity as allelopathic agents. It was reported that *I. cylindrica* may alter the microbial community in invaded soil and enhance the decomposition process of plant residues [54]. The rhizobium nodulation and mycorrhizal colonization of native plant species were significantly lower in *I. cylindrica*-infested plantations [59]. The leachates and exudates of *I. cylindrica* suppressed the mycorrhizal colonization of *Aristida stricta* Michex. *var. beyrichiana* and *Pinus elliottii* Engelm [66]. In addition, aqueous extracts of *I. cylindrica* shoots suppressed the rhizobium nodulation and arbuscular mycorrhizal colonization of *Vigna radiata* (L.) Wilczek and *Phaseolus vulgaris* L. [77]. These observations suggest that certain compounds of *I. cylindrica* may be involved in the alteration of the microbial community, including rhizobia and mycorrhizal fungi. Symbiosis with arbuscular mycorrhizal fungi is important for most territorial plants, and rhizobium nodulation is also important for leguminous plants. Arbuscular mycorrhizal fungi increase the ability of plants to absorb water and nutrients and enhance the defense function against several stress conditions and pathogen attacks [100,101,102]. Rhizobia establish root nodules of legumes, fix nitrogen and supply ammonium to host plants [103,104]. These observations suggest that *I. cylindrica* may be able to degrade the fungal and bacterial mutualisms of nearby plants. Reductions in rhizobium nodulation and mycorrhizal colonization weaken the ability of native plants to absorb water and nutrients, as well as their defense functions, which may result in growth inhibition of the native plants. It was also reported that other invasive plant species, such as *Lantana camara* L. and *Fallopia* *japonica* (Houtt.) Ronse Decraene, alter the microbial community, which is involved in the mycorrhizal colonization and the decomposition process of plant residues [105,106,107,108,109,110]. As described above, *I. cylindrica* may directly interrupt the regeneration process of native plant species by decreasing their germination and growth and indirectly via the suppression of mycorrhizal colonization and rhizobium nodulation of native plant species through its allelopathy. Therefore, the allelopathy of *I. cylindrica* may contribute to its enhanced competitive ability, as well as its invasiveness (Figure 3). Catechin (flavonoid) was once considered to contribute to the invasion of *Centaurea stoebe* L. from Europe into North America [49,50]. According to the novel weapon hypothesis, *C. stoebe* would release a certain amount of catechin into the rhizosphere soil, and the released catechin could suppress the regeneration process of indigenous plant species in North America through the inhibition of their germination and growth, contributing to the formation of *C. stoebe* monospecific stands [50,51]. However, the actual catechin levels in the rhizosphere soil were very low and could not induce significant inhibition of indigenous plant species [111]. The consequence suggests that the levels of allelochemicals in the rhizosphere soil and/or surrounding environments of invasive plants should be determined. Some allelochemicals were found in leachates, root exudate and growth medium of *I. cylindrica* [64,66,67]. However, it was also reported that some allelochemicals were absorbed onto soil particles. Some of those allelochemicals were not liberated with soil water and could not work as allelopathic agents [112,113,114].

Therefore, a discussion of the specific inhibitory activity of the identified allelochemicals and their concentrations in the rhizosphere soil water and/or surrounding environments is necessary to evaluate the contribution of these allelochemicals to the invasiveness of *I. cylindrica*.

## 6. Conclusions

*I. cylindrica* is highly invasive and has been naturalized in humid tropics, subtropics and warmer temperate zones of the world. Large monospecific stands of *I. cylindrica* are often observed in several countries. Its characteristics of life history, such as high reproduction and growth rates, competitive ability, phenotypic plasticity and defense ability against natural enemies may contribute to the invasiveness of the species. It was also shown that *I. cylindrica* possesses allelopathic properties (Table 1) and contains allelochemicals (Table 2, Figure 2), some of which were found in leachates, root exudates and growth medium of *I. cylindrica,* suggesting that certain allelochemicals may be released into the rhizosphere soil and/or surrounding environments. The leachates, exudates and extracts of *I. cylindrica* also altered the microbial community and suppressed the rhizobium nodulation and mycorrhizal colonization of native plant species, suggesting that certain compounds of *I. cylindrica* may be involved in the alteration of the microbial community, including rhizobia and mycorrhizal fungi. Mycorrhizal colonization increases the ability of plants to absorb water and nutrients and enhances the defense ability against several stress conditions and pathogen attacks. Rhizobia induce the root nodulation of legumes and supply nitrogen to the host plants. Therefore, the suppression of rhizobium nodulation and mycorrhizal colonization reduces the competitive ability and vigor of native plant species. Allelochemicals of the species may also suppress the regeneration process of native plant species through the inhibition of their germination and growth. Therefore, allelochemicals released from *I. cylindrica* may provide the species with a competitive advantage against native plant species, interrupting the regeneration process of native plants. The allelopathy of *I. cylindrica* may contribute to its invasiveness and naturalization as an invasive plant species.

## Figures and Tables

**Table 1:** Allelopathic activities of leachates, exudates, residues, soil and plant extracts of *Imperata cylindrica*.

Source	Inhibition			Target Plant Species	Reference
	Germination	Growth	Mycorrhizal colonization, nodulation		
**Leachate**		✓		*Brachiaria mutica, Digitaria decumbens*	[64]
	✓	✓		*Dichanthium annulatum*	[65]
		✓		*Chrysopogon montanus, Medicago polymorpha, Pinus roxburghii*	[65]
**Leachate/** **exudate**		✓	✓	*Aristida stricta*	[66]
**Exudate**			✓	*Pinus elliottii*	[66]
		✓		*Echinochloa crus-galli*	[67]
**Plant residue**		✓		*Erigeron candensis, Portulaca oleracea*	[68]
	✓	✓		*Sida spinosa, Brachiaria ramosa, Echinochloa crus-galli, Lolium multiflorum*	[69]
	✓			*Lolium multiflorum*	[69]
		✓		*Oryza sativa*	[70]
		✓		*Trifolium subterraneum*	[71]
**Soil extract**	✓	✓		*Dichanthium annulatum, Chrysopogon montanus*	[65]
		✓		*Setaria italica, Medicago polymorpha, Pinus roxburghii*	[65]
**Plant extract**	✓			*Oryza sativa*	[70]
	✓	✓		*Parthenium hysterophorus*	[72]
	✓	✓		*Cucumis sativus, Lolium perenne*	[73]
	✓	✓		*Centrosema pubescens*	[74]
	✓	✓		*Raphanus sativus, Brassica juncea, Trigonella foenum-graecum, Lycopersicon esculentum*	[59]
		✓		*Solanum lycopersicum (syn; L.esculentum)*	[75]
	✓	✓		*Sida spinosa, Brachiaria ramosa, Echinochloa crus-galli, Cynodon dactylon, Lolium multiflorum*	[76]
	✓	✓		*Setaria italica, Dichanthium annulatum, Chrysopogon montanus, Medicago polymorpha, Pinus roxburghii*	[65]
			✓	*Vigna radiata, Phaseolus vulgaris*	[77]
	✓			*Echinochloa crus-galli, Lolium multiflorum, Phleum pratense, Lepidium sativum, Lactuca sativa, Medicago sativa*	[78]
	✓			*Amaranthus spinosus*	[79]
	✓	✓		*Lactuca sativa, Agrostis stolonifera*	[80]

**Table 2:** Allelochemicals identified in *Imperata cylindrica* and their sources.

Chemical Class	Compound	Source					Reference
		Leachate	Exudate	Essential oil	Rhizome extract	Leaf extract	
Fatty acid	1: Palmitic acid		✓	✓			[67,80]
	2: Linoleic acid				✓		[67]
Terpenoid	3: Phytol			✓			[80]
	4: Tabanone			✓			[80]
Simple phenolic	5: *p-*Vinylguaiacol			✓			[80]
	6: 2,4-Di-*tert*-butylphenol				✓		[67]
Benzoic acid	7: Benzoic acid		✓			✓	[66,75]
Phenolic acid	8: Salicylic acid		✓				[66]
	9: Gentisic acid					✓	[75]
	10: Gallic acid		✓				[66]
	11: *p*-Hydroxybenzoic acid	✓				✓	[64,75]
	13: van ilic acid					✓	[75]
Phenolic aldehyde	14: van illin				✓	✓	[67,75]
	15: *p*-Hydroxybenzaldehyde					✓	[75]
Acetophenone	16: 4-Acetyl-2-methoxyphenol (acetoguaiacone)		✓				[67]
Phenylpropanoid	17: Isoeugenol				✓		[67]
	18: cinnamic acid		✓				[66]
	19: *p*-coumaric acid	✓				✓	[64,75]
	20: *o*-coumaric acid					✓	[75]
	21: Caffeic acid		✓				[66]
	22: Ferulic acid				✓		[67]
	23: Isoferulic acid				✓		[67]
	24: Sinapinic acid (sinapic acid)		✓				[66]
	25: Methyl caffeate				✓		[78]
Flavonoid	26: 5-Methoxyflavone				✓		[78]
	27: 5,2′-Dimethoxyflavone				✓		[78]
Quinone	28: Emodin		✓				[66]
Alkaloid	29: Hexadecahydro-1-azachrysen-8-yl ester		✓				[66]
